# CfDNA methylation profiling in blood following moderate-to-severe traumatic brain injury using brain cell type–enriched signatures: A pilot study

**DOI:** 10.1371/journal.pone.0358763

**Published:** 2026-10-06

**Authors:** Shih-Han Kao, Larry N. Singh, Jonathan Starr, Firas H. Kobeissy, Claudia Robertson, Spyridoula Tsetsou, John B. Williamson, Damon G. Lamb, Amy K. Wagner, Zhifeng Kou, Robert D. Welch, Jose-Miguel Yamal, Luis Leon-Novelo, Richard Rubenstein, Kevin K. W. Wang, Todd J. Kilbaugh

**Affiliations:** 1 Resuscitation Science Center of Emphasis, Department of Anesthesiology and Critical Care Medicine, The Children’s Hospital of Philadelphia, Philadelphia, Pennsylvania, United States of America; 2 Center for Neurotrauma, Multiomics & Biomarkers, Department of Neurobiology, Neuroscience Institute, Morehouse School of Medicine, Atlanta, Georgia, United States of America; 3 Department of Neurosurgery, Baylor College of Medicine, Houston, Texas, United States of America; 4 Department of Neurology, Baylor College of Medicine, Houston, Texas, United States of America; 5 Brain Rehabilitation Research Center, Malcom Randall VA Medical Center, Gainesville, Florida, United States of America; 6 Department of Psychiatry, University of Florida, College of Medicine, Gainesville, Florida, United States of America; 7 Department of Physical Medicine & Rehabilitation, University of Pittsburgh, Pittsburgh, Pennsylvania, United States of America; 8 Safar Center for Resuscitation Research, University of Pittsburgh, Pittsburgh, Pennsylvania, United States of America; 9 Department of Neuroscience, University of Pittsburgh, Pittsburgh, Pennsylvania, United States of America; 10 Center for Neuroscience, University of Pittsburgh, Pittsburgh, Pennsylvania, United States of America; 11 Clinical and Translational Science Institute, University of Pittsburgh, Pittsburgh, Pennsylvania, United States of America; 12 Department of Biomedical Engineering, New Jersey Institute of Technology, Newark, New Jersey, United States of America; 13 Department of Emergency Medicine, Wayne State University School of Medicine, Detroit, Michigan, United States of America; 14 Department of Biostatistics and Data Science, School of Public Health, The University of Texas Health Science Center, Houston, Texas, United States of America; 15 Department of Neurology, SUNY Downstate Health Sciences University, Brooklyn, New York, United States of America; 16 Translational and Multi-Omic Medicine, Foundation for Applied Molecular Evolution, Alachua, Florida, United States of America; 17 Department of Anesthesiology and Critical Care Medicine, Perelman School of Medicine at the University of Pennsylvania, Philadelphia, Pennsylvania, United States of America; Southern Illinois University Carbondale, UNITED STATES MINOR OUTLYING ISLANDS

## Abstract

The human brain consists of diverse cell types that collaboratively perform complex functions and form intricate neuronal networks. When traumatic brain injury (TBI) occurs, multiple molecular pathways involving diverse cell types are activated in response to injury. Understanding how brain cell composition is altered during TBI may help improve patient outcomes. Upon brain trauma, neurovascular disruption leads to the release of brain-derived cell-free DNA (cfDNA) into peripheral blood. The tissue-specific DNA methylation patterns in this cfDNA have the potential to serve as candidate markers for studying brain injury. To characterize brain cell type–enriched methylation features, we analyzed the methylomes of five major brain cell types—neurons, astrocytes, oligodendrocytes, microglia, and vascular endothelial cells (VECs)—using whole-genome bisulfite sequencing (WGBS). Cellular enrichment analysis showed that differentially methylated genes (DMGs) exhibited reference-defined cell type–enriched patterns. Gene Ontology pathway analysis further supported the biological plausibility of these methylation features. To demonstrate the feasibility of applying cfDNA methylation profiling in TBI, we analyzed a limited pilot dataset comprising healthy controls and samples collected at acute (1 day), subacute (1 month), and chronic (6 months) post-injury time points from patients with moderate-to-severe TBI (msTBI). These analyses identified candidate brain cell type–enriched cfDNA methylation features that differed across post-injury sampling groups. Together, our findings highlight that brain cell type-enriched methylation signatures may provide a powerful tool for characterizing distinct cell populations and nominating candidate brain cell-associated cfDNA methylation biomarkers in TBI.

## Introduction

DNA methylation provides a powerful approach for inferring the cellular composition of tissues during injury. It is a common form of epigenetic modification that predominantly occurs at the cytosine residues preceding guanine (CpG) nucleotides and plays key roles in gene regulation, development and genomic stability [1]. Importantly, DNA methylation also serves as a signature of cell identity as each cell type exhibits distinct methylation patterns [2].

Building on this principle, a variety of computational approaches have been developed to identify differentially methylated regions (DMRs) across biological conditions, such as health versus injury or pre- and post-treatment states [3]. In general, these methods quantify methylation at individual CpG sites, aggregate signals across genomic regions, and then apply statistical models to detect stretches of CpGs that differ significantly between groups [4–8]. Once identified, these DMRs can be leveraged for biomarker discovery by linking methylation patterns to disease states. For example, in oncology, tumor-specific DMRs have been used to detect cancer types and estimate tumor burden [9,10], demonstrating the clinical utility and real-world impact of methylation-based biomarkers.

Recently, several computational tools aimed to estimate the cell type composition of bulk DNA samples based on methylation signatures, have been developed for methylation deconvolution [2,11–15]. Linear regression, constrained optimization, or Bayesian inference, can be used to resolve bulk methylation signals into estimated proportions of each cell type. One such method, Unmethylated, Mixed, Methylated (UXM) is a reference-based, fragment-level deconvolution algorithm for DNA methylation sequencing data [2]. It estimates the relative proportions of different cell types in a mixed sample by calculating the percentage of unmethylated fragments per genomic marker using non-negative least squares regression (NNLS) model. The output can be used for tracking cell type-specific injury, inferring cell-free DNA (cfDNA) origins, or characterizing cellular heterogeneity in tissues.

Despite these advances, applying such approaches to traumatic brain injury (TBI) remains challenging due to the brain’s cellular heterogeneity. The human brain comprises diverse cell types organized into multi-layered structures [16], whose interactions regulate functions ranging from basic physiological processes to higher-order cognition [17]. Following TBI, the disruption of tightly coordinated cellular processes triggers a cascade of molecular events across diverse brain cell types, such as neuronal injury signaling, microglial activation, and astrocytic stress responses, complicating both prognosis and treatment [18,19]. As such, unveiling brain cell type-specific contributions is pivotal to improving outcomes for patients with TBI.

Here, we report a pilot study using both a DMR tool and deconvolution algorithm to characterize five brain cell types: neurons, astrocytes, microglia, oligodendrocytes, and vascular endothelial cells (VECs). We also included a cfDNA dataset from 16 individuals with moderate-severe TBI (msTBI) to visualize the methylation changes during acute, subacute, and chronic phases of msTBI. We applied the Azimuth Cell Type database (https://azimuth.hubmapconsortium.org/) and GO enrichment analysis to validate the cell type DMRs. We combined these tools to assess methylation changes in human msTBI cfDNA in order to identify potential brain cell type biomarkers. To our knowledge, this is the first study to systematically interrogate the brain cell-type DNA methylome in the context of human TBI compared with control blood samples.

## Materials and methods

### Patients

TBI severity was defined according to the U.S. Department of Defense (DoD) criteria and guidelines [20]. The study cohort included uninjured controls (N = 5) and individuals with msTBI enrolled at acute (1-day post-injury; N = 4), subacute (1-month post-injury; N = 12), and chronic (6-month post-injury; N = 4) time points. All clinical studies were approved by the relevant local ethics committees as follows: the Ethics Committee at University of Pittsburgh (UPitt) (IRB ID: STUDY22100119; date of recruitment: 06/08/2023− 09/03/2025) and Baylor College of Medicine (BCM) Institutional Review Board (protocol H-48245) and Harris Health system (protocol ID 22-12-2966; date of recruitment: 11/01/2022-10/31/2027). Written informed consent was obtained from all participants or their legally authorized representatives prior to enrollment. Additional patient information is provided in S1 File.

### Cells

Human primary astrocytes (HMP202) and induced pluripotent stem cell (iPSC)-derived astrocytes (40HU-008) were obtained from Neuromics Inc. (Minneapolis, MN) and from iXCells Biotechnologies (San Diego, CA), respectively. Human glial cell line SVG p12 cells (CRL-8621) and peripheral blood mononuclear cells (PBMCs, PCS-800–011) were obtained from ATCC (Manassas, VA), and whole blood cells were provided by the Coriell Institute for Medical Research (Camden, NJ). VECs (HEC02) were obtained from Neuromics (Minneapolis, MN), Cell Systems (ACBRI 376, Kirkland, WA), ScienCell (1009, Carlsbad, CA), iXCells (10HU-051, San Diego, CA), and AcceGen (ABC-TC3524, Fairfield, NJ). Primary microglia cells (1909) were purchased from ScienCell (Carlsbad, CA). The HOG oligodendrocyte cell line (SCC163) was obtained from Sigma-Aldrich (St. Louis, MO), and human SH-SY5Y neuroblastoma cells were generously provided by Dr. Kevin Wang (originally from ATCC). All cell lines were authenticated by STR profiling and tested negative for mycoplasma contamination prior to use. One control cfDNA sample was obtained from Biochain (Newark, CA). Raw human neuron and oligodendrocyte primary cell data were downloaded from the Gene Expression Omnibus (GEO) GSE107729. Genomic DNA (gDNA) was extracted using DNeasy Blood and Tissue Kits (Qiagen, Germantown, MD) and was subsequently subjected to bisulfite conversion and WGBS (see section below).

### Blood collection & cfDNA extraction

Blood was drawn at UPitt and BCM into the cf-DNA/cf-RNA Preservative Tubes (Norgen Biotek, Ontario, Canada). Plasma was separated by centrifugation at 1,600 x g at each clinical site and stored at −80 °C. The samples were shipped on dry ice to the analytical site (Children’s Hospital of Philadelphia) and stored at −80 °C until use. Prior to extraction, samples were thawed on ice and then centrifuged at 16,000 x g. The cfDNA was extracted using the Chemagic 5k cfDNA kit (Revvity Health Sciences, Hopkinton, MA) and quantified using the Qubit 1x dsDNA high sensitivity assay kit (Thermo Fisher Scientific, Waltham, MA) on a Qubit 4 Fluorometer (Thermo Fisher Scientific). The cfDNA fragment sizes were assessed with the cell-free DNA ScreenTape assay on the Agilent 4200 TapeStation system (Agilent Technologies, Santa Clara, CA).

### Bisulfite conversion, sequencing, and data processing

All DNA samples were subjected to bisulfite conversion using the Zymo Bisulfite conversion kit (Zymo Research, Irvine, CA). WGBS libraries were prepared from the bisulfite-converted samples using the xGen Methylation-Sequencing DNA Library Preparation Kit (Integrated DNA Technologies, Coralville, IA) according to the manufacturer’s instructions. Sequencing was performed on the NovaSeq 6000 platform (Illumina, San Diego, CA) to generate 2 × 150 bp paired-end reads at approximately 30 × coverage. Raw reads were demultiplexed, quality assessed using FastQC (v0.12.1), trimmed with Trim Galore (v0.6.10), and aligned to the human reference genome (GRCh38/hg38) using Bismark (v0.24.2) [21]. Bismark was also used for PCR deduplication, post-alignment quality control, and methylation extraction to obtain CpG methylation calls for each sample.

### Differential methylation region (DMR) analysis

To identify the cell type of origin of cfDNA, bisulfite-treated reads were mapped and methylation calls were generated from the sequencing data using Bismark [21]. Bismark coverage files were imported into SeqMonk (RRID:SCR_001913; https://www.bioinformatics.babraham.ac.uk/projects/seqmonk/) using the “Import Data” and “Bismark (Cov)” options, where the methylation quantitation pipeline enables analysis across custom-defined regions [8,22,23]. For CpG coverage analysis, probes were generated using the “Read position Probe Generator.” CpGs with a minimum of 1 read were included in the 5x minimum threshold analysis. Methylation values for each probe were quantified using the “Bisulphite methylation over features” pipeline.

To create the brain cell type methylation atlas, brain cell type-enriched DMRs were selected based on the following criteria: Significant differences between each brain cell type and blood were assessed using Chi-square tests with false discovery rate (FDR) correction, applying a significance threshold of FDR < 1 × 10^−10^. A DMR was defined as cell type-enriched if it showed a methylation level difference of >25% in the respective cell type compared to others, while the same region exhibited methylation levels within either the unmethylated (0–20%) or methylated (80–100%) range in the other four brain cell types and in blood. Cell type-enriched DMRs were exclusive to their respective brain cell types and were not identified as enriched in the other four cells and blood (*e.g.*, neuron-enriched DMRs were not enriched in astrocytes, microglia, oligodendrocytes, VECs, or blood).

The cfDNA methylation profiles were compared with the brain cell type methylation atlas described above to infer the contribution of brain-derived signals in acute to chronic msTBI cfDNA samples. A cell type-enriched TBI DMR was defined as a region exhibiting the characteristic methylated or unmethylated pattern of the corresponding cell type, while the same region in control cfDNA displayed the opposite methylation state. Differentially methylated genes (DMGs) were defined as genes whose entire gene bodies were precisely overlapped by the start and end coordinates of a DMR. The brain cell type double-dendrogram heatmap was constructed using methylation values. Only features meeting a false discovery rate (FDR) threshold of < 1 × 10^−10^ were included in the visualization. Unsupervised hierarchical clustering was performed using Euclidean distance and complete linkage. Cell type-enriched DMG heatmaps were used to visualize methylation patterns across cell types. Methylation values were z-score normalized across samples prior to visualization.

### Cell type and functional enrichment analysis

We analyzed cell type enrichment of DMGs using Azimuth, a pre-annotated single-cell RNA-seq reference atlas for human tissues, and performed functional enrichment analysis using Gene Ontology in ShinyGO (v0.80) [24]. Homo sapiens (GRCh38.p13) was selected as the genome assembly. Azimuth cell type terms and Gene Ontology terms from Biological Processes (BP) and Cellular Components (CC) categories with an FDR < 0.05 were considered significantly enriched. Enrichment of DMGs was ranked primarily by fold enrichment, followed by FDR and the number of genes.

### Deconvolution analysis

The UXM deconvolution algorithm was obtained from https://github.com/nloyfer/UXM_deconv. A reference atlas was rebuilt using WGBS data from the brain cell types listed in Table 1 while retaining the leukocyte cell data included in UXM. The cfDNA methylome data were then imported. All the Binary Alignment/Map (BAM) files were converted to  .pat format, and sample groups were generated accordingly.

**Table 1:** Brain and blood cell datasets.

Cell Type	N	Source	Platform	Accession
neurons	26	primary cells, immortalized cancer cell line	WGBS	GSE107729
astrocytes	4	primary cells, iPSC-derived cells, immortalized cells, immortalized cancer cell line	WGBS	Pending (to be deposited)
microglia	1	primary cells	WGBS	Pending (to be deposited)
oligodendrocytes	21	primary cells, immortalized cancer cell line	WGBS	GSE107729
VECs	6	primary cells	WGBS	Pending (to be deposited)
blood cells	4	whole blood, PBMC	WGBS	Pending (to be deposited)
Total	62			

^a^N = Number of samples.

### Statistical analysis

For DMR analysis, chi-square tests were performed using the SeqMonk package, with the Benjamini—Hochberg procedure applied to control the FDR. Differences were considered significant at FDR < 1 × 10^−10^.

## Results

### Patient description

We enrolled 16 individuals with TBI and 5 uninjured controls. Four individuals with TBI had blood samples collected at both 1 month and 6 months post-injury. The cohort had a median age of 58 years at enrollment, 81% were male, 90.4% White, 9.6% Hispanic, and 23.8% had severe TBI (Table 2).

**Table 2:** Demographics of the sample population.

	UPITT (N = 12)	BCM (N = 4)	Control (N = 5)^a^	Total (N = 21)
**Age (years)**				
Median (Q1, Q3)	57	44.5	64.5	58
	(48.0, 65.8)	(33.5, 57.8)	(60.0, 68.8)	(48.0, 68.8)
**Biological sex, n (%)**				
Male	10 (83.3%)	4 (100.0%)	3 (60.0%)	17 (81.0%)
Female	2 (16.7%)	0 (0.0%)	1 (20.0%)	3 (14.3%)
Unknown	0 (0.0%)	0 (0.0%)	1 (20.0%)	1 (4.7%)
**Race, n (%)** ^**b**^				
White	11 (91.7%)	4 (100.0%)	4 (80.0%)	19 (90.4%)
Black or African-American	1 (8.3%)	0 (0.0%)	0 (0.0%)	1 (4.8%)
Unknown	0 (0.0%)	0 (0.0%)	1 (20.0%)	1 (4.8%)
**Ethnicity, n (%)**				
Hispanic or Latino	0 (0.0%)	2 (50.0%)	0 (0.0%)	2 (9.6%)
Not Hispanic or Latino	12 (100.0%)	2 (50.0%)	4 (80.0%)	18 (85.7%)
Unknown	0 (0.0%)	0 (0.0%)	1 (20.0%)	1 (4.7%)
**GCS (TBI only), n (%)**				
13-15	5 (41.7%)	4(100%)	—	9 (56.2%)^c^
9-12	2 (16.6%)	0 (0.0%)	—	2 (12.5%)^c^
3-8	5 (41.7%)	0 (0.0%)	—	5 (31.3%)^c^

^a^Four healthy control samples obtained from UPitt. One healthy control sample purchased from Biochain that has no demographic information.

^b^No Asian, Native Hawaiian or Other Pacific Islander, American Indian or Alaska Native.

^c^Percentages for GCS are calculated among TBI participants only (N = 16). GCS was not assessed in controls.

### The DMR analysis in human brain cell types

In this pilot study, we established a human brain cell type methylation atlas using DNA methylomic data from neurons, astrocytes, microglia, oligodendrocytes, and VECs (Table 1). The reference dataset included primary cells, iPSC-derived cells, and immortalized cell lines. Since blood cells are the major contributors to cfDNA in peripheral blood [25–27], WGBS data from blood were included as background. As a small test set, we analyzed cfDNA samples collected at 1 day (N = 4), 1 month (N = 12), and 6 months (N = 4) post-msTBI (Table 2). Control cfDNA was obtained from healthy subjects. We found that the proportion of reads overlapping brain-enriched DMRs was comparable between the TBI (median: 0.248%; IQR: 0.233%–0.255%) and control groups (median: 0.250%; IQR: 0.238%–0.254%), indicating that the overall representation of reads at these loci did not differ substantially between groups (S2 File). However, read overlap alone does not establish that a fragment originated from the brain because it does not account for the fragment’s methylation pattern. Therefore, we further evaluated methylation levels within these brain-enriched DMRs to identify fragments exhibiting brain-enriched methylation signatures and assess injury-associated differences between TBI and control samples.

To identify genomic regions with significant methylation differences between cell types, we performed probe-level methylation analysis by quantifying methylation at each region and applying chi-square tests with multiple-testing correction (Methods; Fig 1A). DMRs were subsequently annotated to their associated genes to derive DMGs, which were further classified as cell type–enriched methylated or unmethylated DMGs.

**Fig 1:**
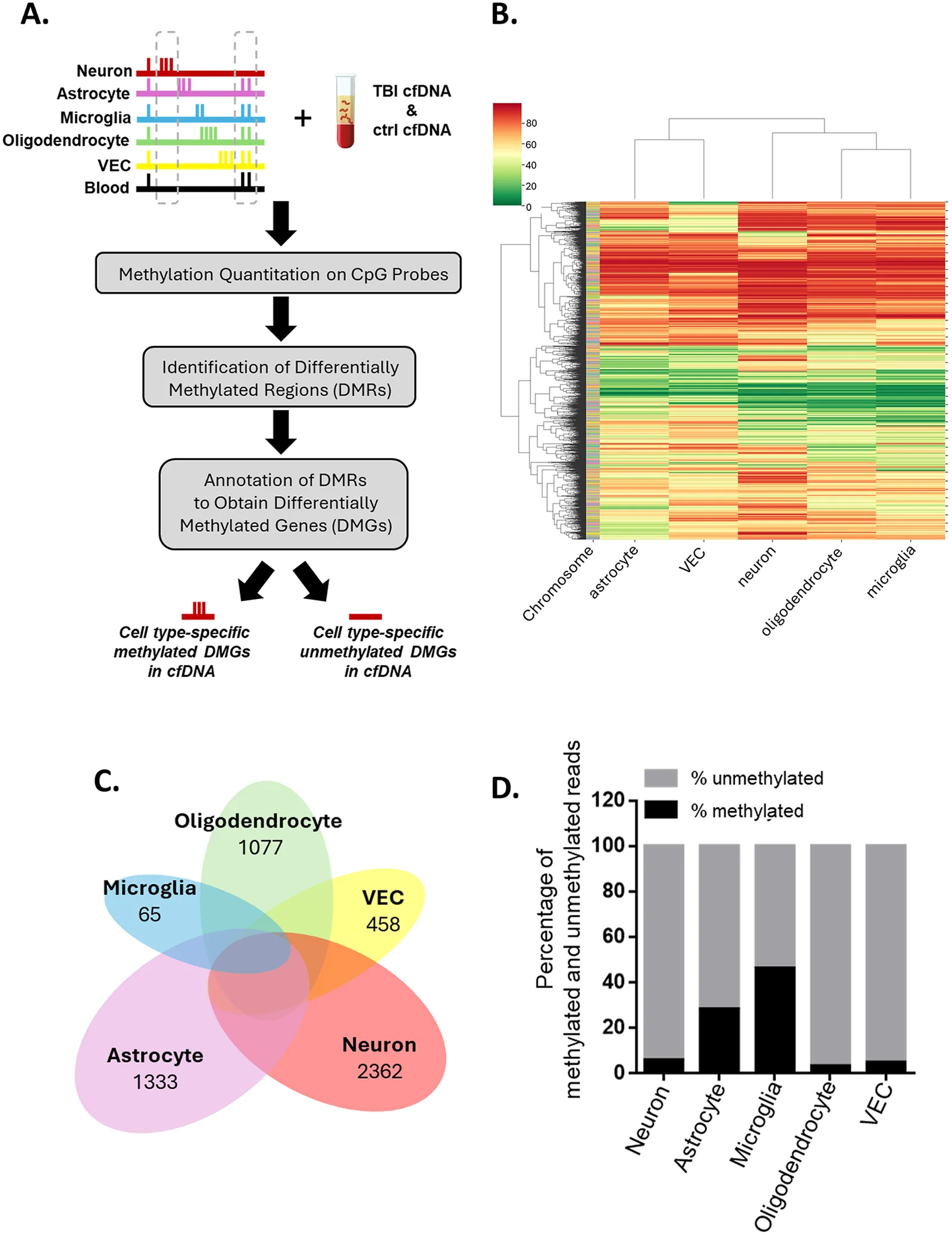
DMR analysis of five human brain cell types. (A) Workflow of probe-level methylation analysis. WGBS data from five human cell types: neurons, astrocytes, microglia, oligodendrocytes, VECs were obtained from the GEO repository or generated in-house. The cfDNA from msTBI patients or healthy controls was also subjected to WGBS. Methylation calls were derived from  .cov files with Bismark and quantified using the bisulfite methylation-over-features pipeline in SeqMonk. Gray dashed lines indicate cell type-enriched DMRs (example shown for neurons). (B) Heatmap with dendrograms showing clustering of DMRs on the vertical axis and cell types on the horizontal axis. CpG probes from reference cell types were combined with those from cfDNA. After methylation quantification, DMRs were clustered by methylation values across cell types and DMR locus. The colors in the heat map represent methylation values. The colors in the chromosome scale represent the chromosomes corresponding to each DMR locus. (C) Venn diagram illustrating the number of cell-type enriched DMRs exclusive to their respective brain cell types. (D) Methylated vs. unmethylated ratios across cell type DMRs.

We performed two-dimensional hierarchical clustering analysis to examine how methylation signatures shape relationships among brain cell types. The analysis was restricted to DMRs that were statistically significant when each brain cell type was compared with blood. The analysis revealed that DMRs from oligodendrocytes and microglia clustered closely (Fig 1B), consistent with their shared glial identity and roles in neuronal support and synaptic plasticity [28,29]. These shared methylation features were followed by neurons, which clustered more distantly from the glial cells. DMRs from astrocytes and VECs formed a separate cluster, consistent with their roles in vascularization, metabolism, and blood-brain barrier (BBB) formation [30–32].

Next, we applied additional criteria to refine the cell type–enriched DMRs, retaining only those exclusive to their respective brain cell types (Methods). A Venn diagram illustrates the number of cell type–enriched DMRs identified (Fig 1C). Most of the cell type-enriched DMRs identified were unmethylated across brain cell types (neurons: 94.2%, astrocytes: 71.7%, microglia: 53.8%, oligodendrocytes: 74.3%, VECs: 95.2%) (Fig 1D). These data suggest that unmethylated regions may serve as robust markers for distinguishing cell identities within the brain.

### Cell type and pathway analysis of cell type-enriched DMGs

#### Neuron-enriched DMGs.

To independently verify the cell type enrichment of DMRs, we first annotated each DMR to the human genome (GRCh38/hg38) to derive differentially methylated genes (DMGs). Then, we used Azimuth to determine whether these DMGs aligned with well-curated cell type annotations. Neuron-enriched DMGs mapped predominantly to glutamatergic neurons in the Azimuth database (Fig 2A). To further explore the roles of neuron-enriched DMGs, we performed GO enrichment analyses of biological processes and cellular components. This analysis revealed enrichment of pathways related to nervous system development (Fig 2B) and multiple neuronal components, including synaptic pathways, dendrite formation, and neuron projection (Fig 2C). These results support the neuronal enrichment of the identified DMGs and validate DMR analysis as an approach for identifying neuron-derived methylation signatures.

**Fig 2:**
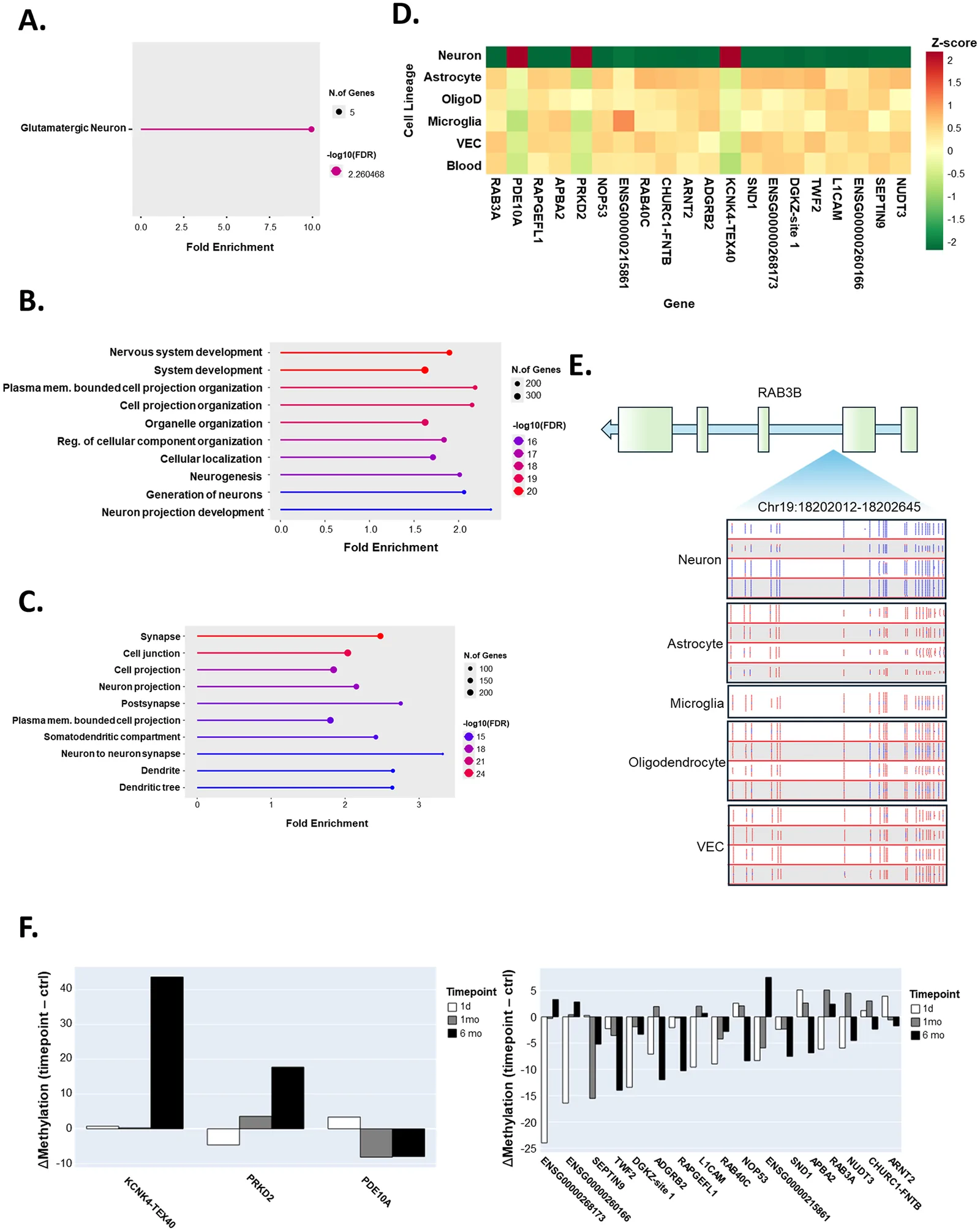
Cell type and pathway analysis of neuron-enriched DMGs with top methylation changes in TBI. (A) Cell type analysis of neuron-enriched DMGs using the Azimuth datasets. (B-C) GO biological process (B) and cellular component (C) enrichment analysis of neuron-enriched DMGs. (D) Heatmap of the top 20 neuron-enriched DMGs. Genes are ranked by the maximum methylation difference between neurons and the other cell types (astrocytes, oligodendrocytes, microglia, VECs, and blood). Values are shown as z-scores computed across cell types. OligoD: oligodendrocytes. (E) Example of bisulfite-converted fragments of a representative gene from four neuron samples, four astrocyte samples, one microglia sample, four oligodendrocyte samples, and four VEC samples. Red and blue dots denote raw methylated and unmethylated CpG counts, respectively. Boxes represent exons and lines represent introns. (F) Methylation changes of the top 20 neuron-enriched DMGs in TBI over time. Neuron-enriched DMGs were classified as methylated (left panel) or unmethylated (right panel) based on their cell lineage-enriched methylation characteristics. Genes were ranked by the maximal direction-consistent absolute methylation change across post-TBI time points (1d, 1mo, 6mo) relative to control.

We next generated a heatmap of the top 20 neuron-enriched DMGs identified in TBI cfDNA. Of these, three (PDE10A, PRKD2 and KCNK4-TEX40) were uniquely methylated in neurons, while the remaining 17 were unmethylated in neurons (Fig 2D). As an example, Fig 2E highlights a neuron-enriched unmethylated locus located in the second intron of *RAB3B* (RAB3B, member RAS oncogene family), a neuronal gene involved in dopamine uptake during synaptic transmission [33]. We profiled methylation changes in the top 20 neuron-enriched DMGs in TBI cfDNA and grouped them according to neuronal methylation signatures (methylated vs. unmethylated; Fig 2F). We found that two of the three methylated neuron-enriched DMGs appeared to exhibit higher methylation levels at 1 month and 6 months post-TBI, whereas most unmethylated neuron-enriched DMGs suggested a trend toward gene-dependent decreases in methylation across post-injury time points, suggesting time point-associated cfDNA methylation patterns among candidate neuronal features. Together, these findings show that neuron-enriched DMGs reflect key characteristics of neuronal cell identity in TBI cfDNA.

#### Astrocyte-enriched DMGs.

To assess the cell type enrichment of astrocyte-enriched DMGs, we queried these genes in the Azimuth database. This analysis showed enrichment for FGFR3 ⁺ , Serpini2 ⁺ layer 1 astrocytes, which ranked as the third most strongly associated cell type in the database (Fig 3A). GO biological process analysis revealed that astrocyte-enriched DMGs are involved in developmental regulation, localization, and cell–cell signaling (Fig 3B). GO cellular component analysis further indicated enrichment in synapse-related pathways and intrinsic components of the plasma membrane (Fig 3C). This finding aligns with the notion that astrocytes actively participate in synaptic function, provide metabolic support, and regulate extracellular K⁺ and Ca² ⁺ levels [34,35].

**Fig 3:**
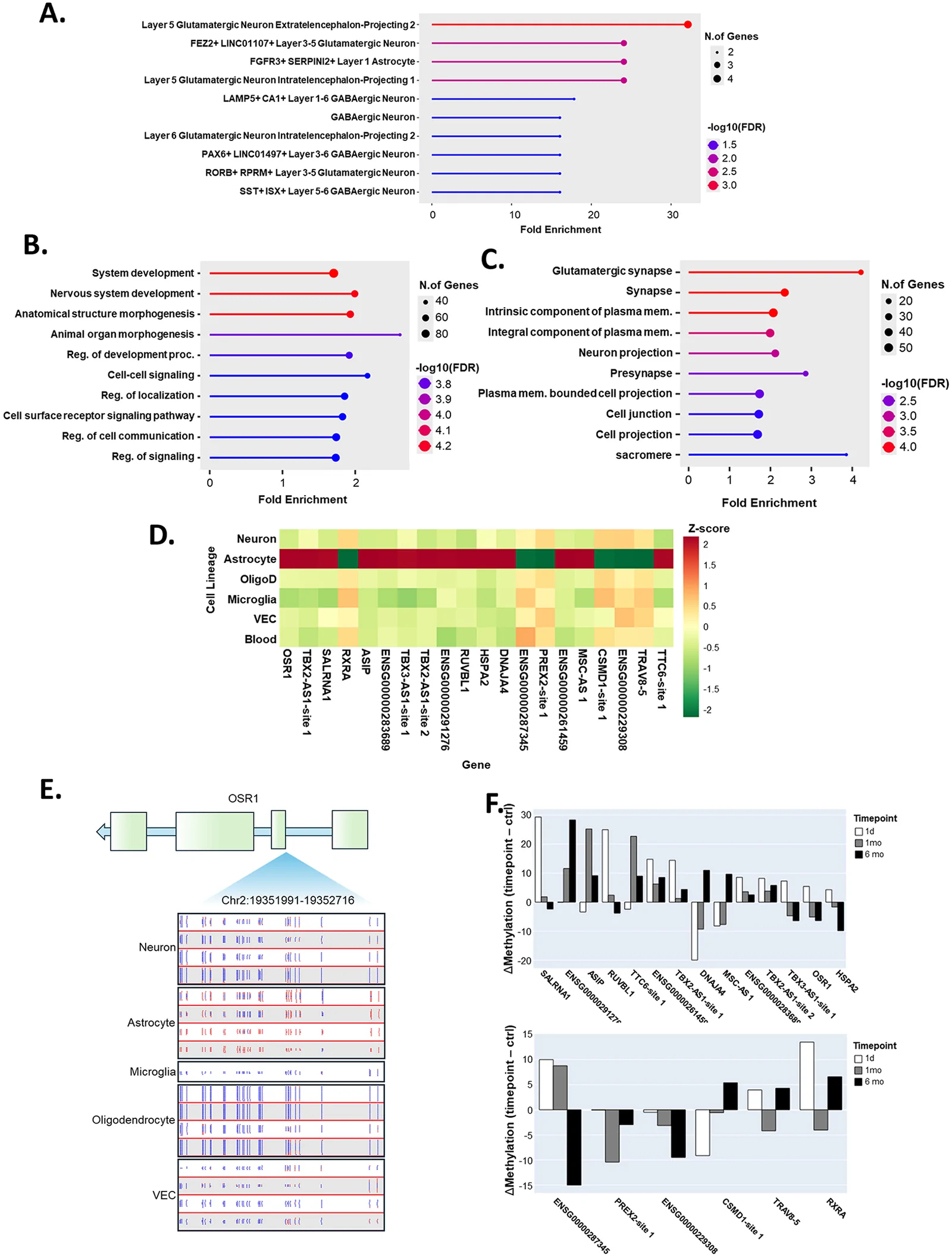
Cell type and pathway analysis of astrocyte-enriched DMGs with top methylation changes in TBI. (A) Cell type analysis of astrocyte-enriched DMGs using the Azimuth datasets. (B-C) GO biological process (B) and cellular component (C) enrichment analysis of astrocyte-enriched DMGs. (D) Heatmap of the top 20 astrocyte-enriched DMGs. Genes are ranked by the maximum methylation difference between astrocytes and the other cell types (neurons, oligodendrocytes, microglia, VECs, and blood). Values are shown as z-scores computed across cell types. OligoD: oligodendrocytes. (E) Example of bisulfite-converted fragments of a representative gene from four neuron samples, four astrocyte samples, one microglia sample, four oligodendrocyte samples, and four VEC samples. Red and blue dots denote raw methylated and unmethylated CpG counts, respectively. Boxes represent exons and lines represent introns. (F) Methylation changes of the top 20 astrocyte-enriched DMGs in TBI over time. Astrocyte-enriched DMGs were classified as methylated (top panel) or unmethylated (bottom panel) based on their cell lineage-enriched methylation characteristics. Genes were ranked by the maximal direction-consistent absolute methylation change across post-TBI time points (1d, 1mo, 6mo) relative to control.

We profiled msTBI cfDNA and identified the top 20 astrocyte-enriched DMGs exhibiting methylation levels in astrocytes that were markedly different from those in other brain cell types. Notably, although most astrocyte-enriched DMRs were unmethylated (Fig 1D), the key regions associated with msTBI in the astrocyte category were predominantly methylated (Fig 3D). As an example, we highlighted the locus-specific methylation profile of *OSR1* (Odd-Skipped Related Transcription Factor 1), an astrocyte-enriched DMG that is highly methylated in astrocytes but unmethylated in other brain cell types (Fig 3E). Methylated astrocyte-enriched DMGs appeared to exhibit higher methylation levels across acute (1 day), subacute (1 month), and chronic (6 months) phases compared with controls (Fig 3F, top). In contrast, unmethylated astrocyte-enriched DMGs appeared to have lower methylation levels during subacute and chronic phases relative to controls, with some gene-specific variability (Fig 3F, bottom). In sum, these findings reveal the distinct astrocytic features of the identified DMGs and time point-associated astrocyte-derived cfDNA methylation signatures following TBI.

#### Microglia-enriched DMGs.

We next assessed the cell specificity of microglia-enriched DMGs. Microglia are immune effector cells in the brain, that perform key functions such as phagocytosis, antigen presentation, and the production of cytokines and chemokines [36]. Consistent with these roles, Azimuth cell type analysis of microglia-enriched DMGs revealed enrichment of several immune-related cell types (Fig 4A), such as EREG+ dendritic cells, plasmacytoid dendritic cells, and CD16 + monocytes. GO enrichment analysis identified only one notable enrichment: the regulation of the laminin-1 binding (Fig 4B). Furthermore, we identified seven microglia-enriched DMGs in msTBI cfDNA, three of which were classified as methylated and four as unmethylated relative to other cell types (Fig 4C). As an example, we demonstrated a microglia-enriched unmethylated locus in VTI1A (Vesicle Transport Through Interaction With T-SNAREs 1A) using raw methylation counts (Fig 4D). When we profiled methylation changes in TBI cfDNA according to microglial methylation signatures, three methylated microglia-enriched DMGs appeared to display higher methylation levels at 1 day or 1 month post-TBI compared to controls (Fig 4E, top). In contrast, four unmethylated microglia-enriched DMGs generally appeared to exhibit lower methylation levels compared to controls across time points, although individual genes showed variable patterns (Fig 4E, bottom). In sum, these findings demonstrate microglia-derived cfDNA methylation characteristics across different time points following TBI.

**Fig 4:**
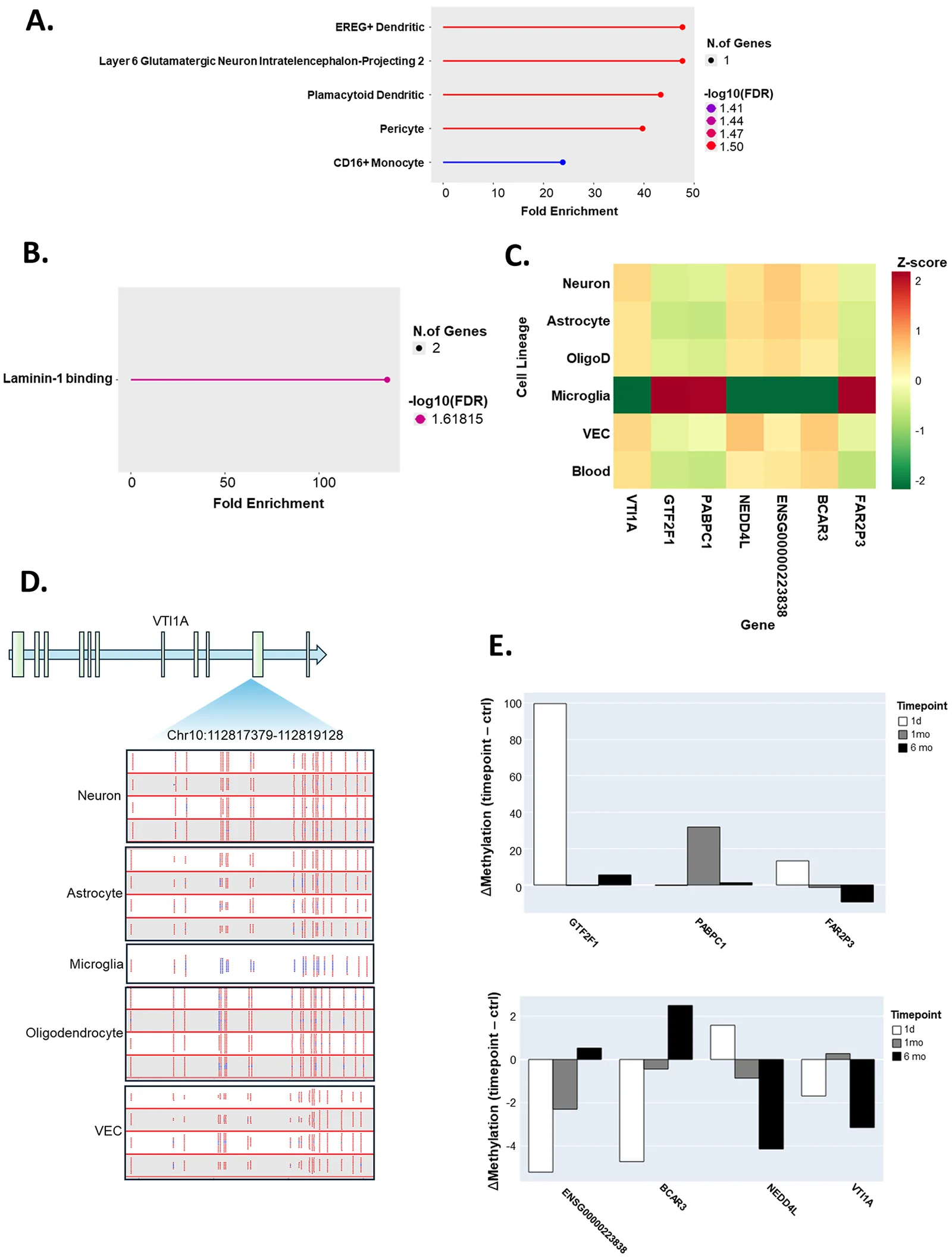
Cell type and pathway analysis of microglia-enriched DMGs with top methylation changes in TBI. (A) Cell type analysis of microglia-enriched DMGs using the Azimuth datasets. (B) GO molecular function enrichment analysis of microglia-enriched DMGs. (C) Heatmap of the top 7 microglia-enriched DMGs. Genes are ranked by the maximum methylation difference between microglia and the other cell types (neurons, astrocytes, oligodendrocytes, VECs, and blood). Values are shown as z-scores computed across cell types. OligoD: oligodendrocytes. (D) Example of bisulfite-converted fragments of a representative gene from four neuron samples, four astrocyte samples, one microglia sample, four oligodendrocyte samples, and four VEC samples. Red and blue dots denote raw methylated and unmethylated CpG counts, respectively. Boxes represent exons and lines represent introns. (E) Methylation changes of the top 7 microglia-enriched DMGs in TBI over time. Microglia-enriched DMGs were classified as methylated (top panel) or unmethylated (bottom panel) based on their cell lineage-enriched methylation characteristics. Genes were ranked by the maximal direction-consistent absolute methylation change across post-TBI time points (1d, 1mo, 6mo) relative to control.

#### Oligodendrocyte-enriched DMGs.

To verify cell type enrichment of oligodendrocyte-associated DMRs, we annotated oligodendrocyte-enriched DMRs to derive oligodendrocyte-enriched DMGs and performed Azimuth cell type analysis. This analysis revealed strong enrichment for oligodendrocytes, followed by oligodendrocyte precursor cells, non-neuronal cells, and PDGFRA + , Col20A1 + , layer 1–6 oligodendrocyte precursor cells (Fig 5A). To explore the functional relevance of these DMGs, we conducted GO enrichment analysis. In the GO biological process category, oligodendrocyte-enriched DMGs were primarily associated with cell development, neurogenesis, and nervous system development (Fig 5B). In the GO cellular component category, the myelin sheath abaxonal region showed the highest enrichment (>20-fold), followed by myelin sheath formation (Fig 5C), consistent with the primary role of oligodendrocytes in myelination and neural support [37,38]. Altogether, these findings indicate that oligodendrocyte-enriched DMGs capture prominent and functionally relevant oligodendrocytic features.

**Fig 5:**
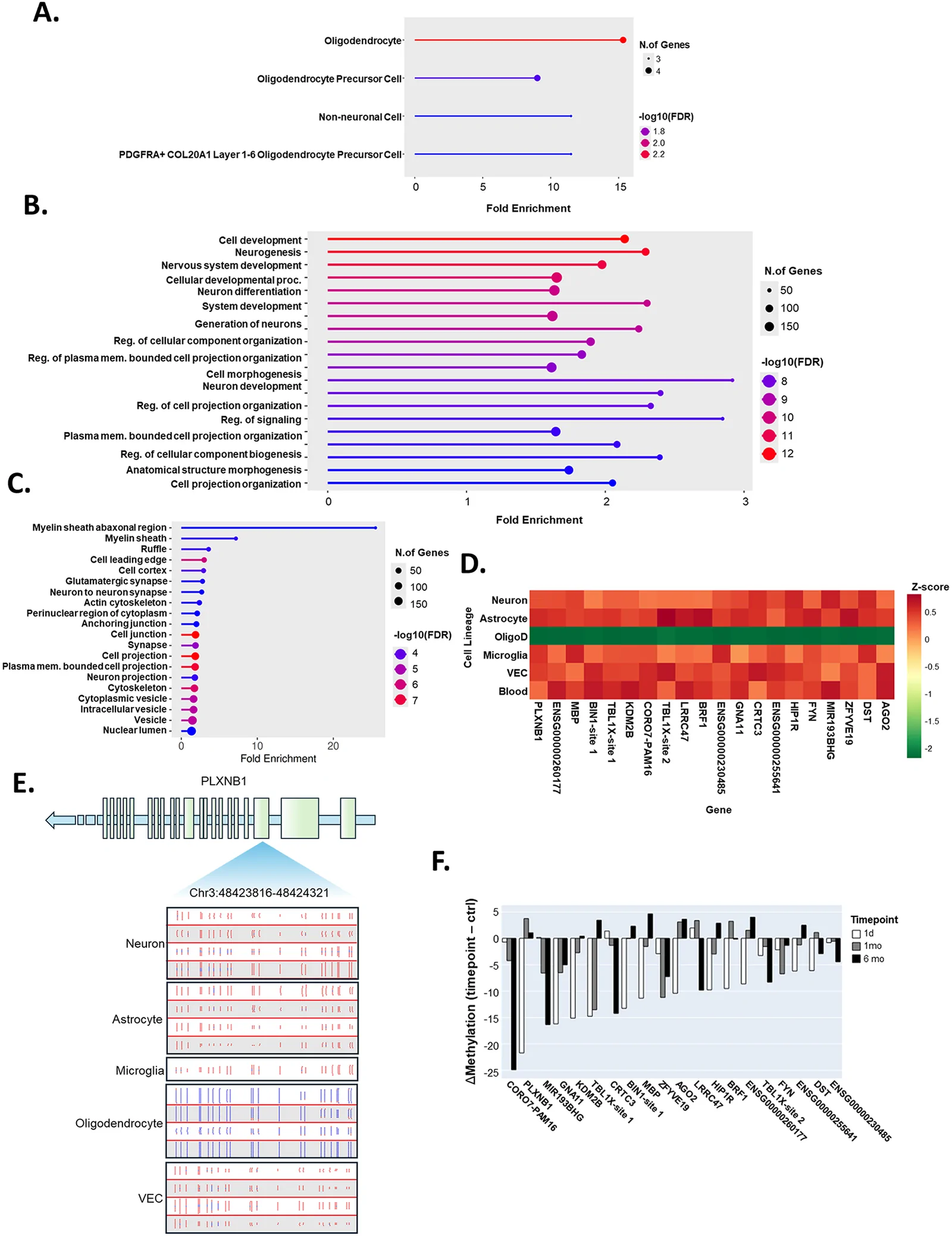
Cell type and pathway analysis of oligodendrocyte-enriched DMGs with top methylation changes in TBI. (A) Cell type analysis of oligodendrocyte-enriched DMGs using the Azimuth datasets. (B-C) GO biological process (B) and cellular component (C) enrichment analysis of oligodendrocyte-specific DMGs. (D) Heatmap of the top 20 oligodendrocytes-enriched DMGs. Genes are ranked by the maximum methylation difference between oligodendrocytes and the other cell types (neurons, astrocytes, microglia, VECs, and blood). Values are shown as z-scores computed across cell types. OligoD: oligodendrocytes. (E) Example of bisulfite-converted fragments of a representative gene from four neuron samples, four astrocyte samples, one microglia sample, four oligodendrocyte samples, and four VEC samples. Red and blue dots denote raw methylated and unmethylated CpG counts, respectively. Boxes represent exons and lines represent introns. (F) Methylation changes of the top 20 oligodendrocytes-enriched DMGs in TBI over time. Oligodendrocytes-enriched DMGs were classified as unmethylated based on their cell lineage-enriched methylation characteristics. Genes were ranked by the maximal direction-consistent absolute methylation change across post-TBI time points (1d, 1mo, 6mo) relative to control.

To highlight the most significant oligodendrocyte-relevant methylation changes during TBI, we present the top 20 oligodendrocyte-enriched DMGs, all of which were uniquely unmethylated compared with other brain cell types (Fig 5D). As a representative example, we display raw methylation counts for one such DMG, *PLXNB1* (plexin B1), across the five brain cell types (Fig 5E). In TBI cfDNA, all 20 unmethylated oligodendrocyte-enriched DMGs generally appeared to exhibit lower methylation levels at at least one post-injury time point relative to controls (Fig 5F). These data highlight time point-associated changes in oligodendrocyte-derived cfDNA signals and suggest white matter involvement across msTBI sampling groups.

#### VEC-enriched DMGs.

Last, we verified the cell specificity of VEC-enriched DMGs using the Azimuth database. Only one cell type, vein, was identified in the cell type analysis (Fig 6A), indicating strong enrichment for this category. To investigate the functional roles of VEC-enriched DMGs, we performed GO pathway enrichment analysis. In the biological process category, VEC-enriched DMGs were enriched in pathways related to anatomical structure morphogenesis, cellular and system development, and nervous system development (Fig 6B). In the cellular component category, VEC-enriched DMGs were enriched in pathways related to junctions, adhesion, and neuron projections (Fig 6C). These findings are consistent with the fundamental roles of VECs in providing supportive architectural environments, forming intercellular junctions, and modulating adhesion [39,40].

**Fig 6:**
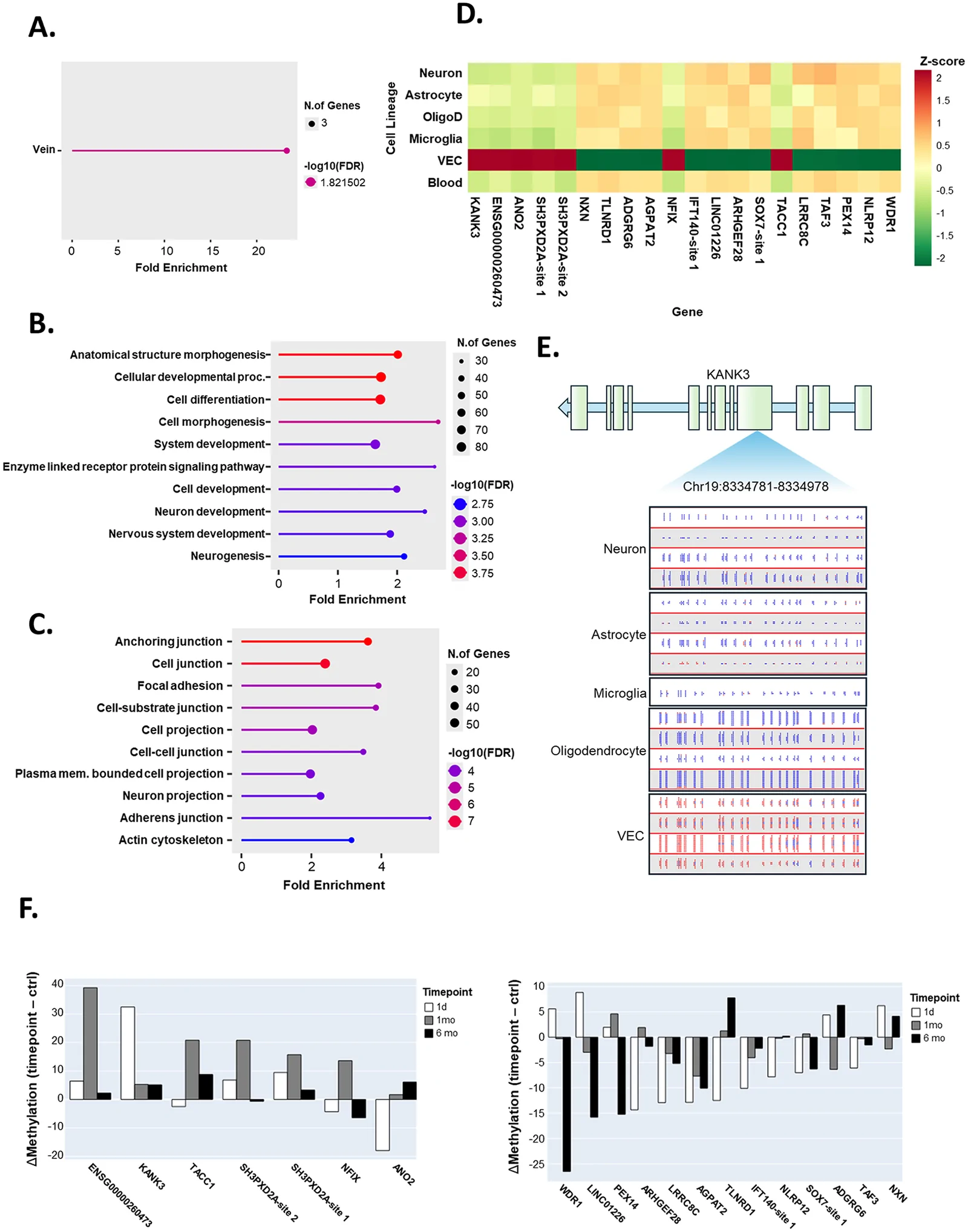
Cell type and pathway analysis of VEC-enriched DMGs with top methylation changes in TBI. (A) Cell type analysis of VEC-enriched DMGs using the Azimuth datasets. (B-C) GO biological process (B) and cellular component (C) enrichment analysis of VEC-enriched DMGs. (D) Heatmap of the top 20 VEC-enriched DMGs. Genes are ranked by the maximum methylation difference between VECs and the other cell types (neurons, astrocytes, oligodendrocytes, microglia, and blood). Values are shown as z-scores computed across cell types. OligoD: oligodendrocytes. (E) Example of bisulfite-converted fragments of a representative gene from four neuron samples, four astrocyte samples, one microglia sample, four oligodendrocyte samples, and four VEC samples. Red and blue dots denote raw methylated and unmethylated CpG counts, respectively. Boxes represent exons and lines represent introns. (F) Methylation changes of the top 20 VEC-enriched DMGs in TBI over time. VEC-enriched DMGs were classified as methylated (left panel) or unmethylated (right panel) based on their cell lineage-enriched methylation characteristics. Genes were ranked by the maximal direction-consistent absolute methylation change across post-TBI time points (1d, 1mo, 6mo) relative to control.

To determine which VEC-enriched DMGs contribute to TBI cfDNA, we profiled the top 20 genes and found that seven were uniquely methylated and 13 were unmethylated in VECs relative to other brain cell types (Fig 6D). As an illustration, Fig 6E shows differential methylation counts for the top-ranked gene, *KANK3* (KN Motif And Ankyrin Repeat Domains 3), at the fourth exon. Methylated VEC-enriched DMGs in TBI cfDNA appeared to exhibit higher methylation levels during the acute (1 day) and subacute (1 month) phases relative to controls (Fig 6F, left). In contrast, unmethylated VEC-enriched DMGs showed a trend toward reduced methylation, with the largest apparent decrease during the chronic phase (6 months), followed by the acute phase, compared to controls (Fig 6F, right). These findings demonstrate the temporal changes in VEC-enriched methylation signatures in TBI cfDNA.

Together, these findings support our DMR analysis and demonstrate the utility of this protocol, which will be further applied and extended in our ongoing study.

### Cell type deconvolution analysis in TBI cfDNA

To quantify overall brain cell type proportions in blood, we also applied the UXM deconvolution algorithm to investigate changes in brain-derived components following msTBI. This analysis included methylome profiles of five brain cell types—neurons, astrocytes, microglia, oligodendrocytes, and VECs—along with blood as a reference. Compared with controls, the overall fraction of brain-derived components was higher at 1-day post-TBI and gradually became lower 1 month and 6 months post-injury (Fig 7), enabling estimation of brain-derived cfDNA contributions following msTBI. Together, these findings reveal the time-associated dynamics of brain-derived cfDNA following TBI, suggesting that cfDNA methylation profiles capture biologically meaningful variation across post-injury phases. Although our present work is primarily cross-sectional with limited paired samples, it establishes a foundation for ongoing longitudinal analyses as additional cases are incorporated.

**Fig 7:**
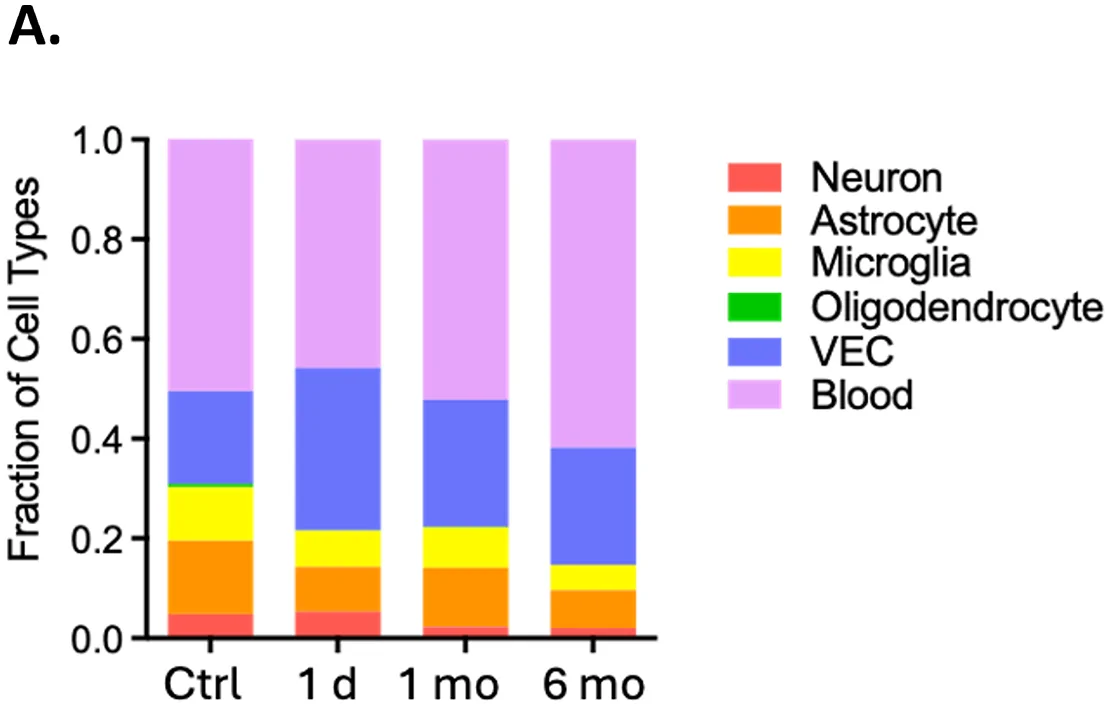
Cell type composition in TBI cfDNA using the UXM deconvolution algorithm. Cell type composition (neurons, astrocytes, microglia, oligodendrocytes, VECs, and leukocytes) in control (ctrl) and TBI cfDNA samples at 1-day (1 d), 1-month (1 mo), and 6-month (6 mo) post-TBI, deconvoluted using the UXM algorithm.

## Discussion

We present a pilot methylomic framework that combines DMR-based analysis with cfDNA deconvolution to characterize candidate brain cell type-enriched cfDNA methylation features across post-injury sampling windows of msTBI. In this framework, DMR analysis was evaluated using independent reference datasets, supporting both the cell type enrichment and functional relevance of cell type–enriched DMGs. Across post-injury sampling windows, several DMGs showed methylation differences in TBI cfDNA that were consistent with reference-defined brain cell type-enriched methylation patterns. Complementing this approach, deconvolution analysis provides a broader view of brain-derived components in blood, supporting the feasibility of using cfDNA methylation profiles to nominate candidate blood-based markers of brain injury for future validation.

While corroborating cell-type enrichment using Azimuth, we observed that the enriched cell types for astrocyte- and microglia-enriched DMGs did not align as closely with the designated categories as those for other cell types. This finding is expected given that Azimuth relies on RNA-seq–derived markers [41], whereas differentially methylated regions (DMRs) do not necessarily correlate one-to-one with transcript abundance. In particular, several exemplar DMGs were located in intronic or exonic regions rather than at transcription start sites (TSSs), where the relationship between methylation and gene expression is less direct [42–44]. We attempted to restrict analyses to DMRs overlapping TSSs; however, the resulting set was too small to support robust cell type enrichment testing.

We found that the majority of cell lineage—enriched DMGs in this small msTBI dataset were hypomethylated. Although DNA methylation patterns at individual loci can vary, prior experimental TBI studies have reported global cellular hypomethylation, particularly in microglia and macrophages [45]. These findings raise the possibility that brain injury may drive broad remodeling of the methylation—demethylation machinery, potentially involving altered expression, enzymatic activity, or subcellular localization of DNA methylation regulators [46–48].

Cell type–enriched DMGs identified in TBI cfDNA may represent candidate biomarkers for future validation and help guide future studies of brain injury biology. While some genes have well-established roles in particular cell types, others are less clearly defined. For instance, we identified *MBP* among oligodendrocyte-enriched DMGs, consistent with its well-known role as a major component of the myelin sheath in oligodendrocytes and Schwann cells [37]. In contrast, *PLXNB1*, which is primarily implicated in GABAergic synapse development [49], was also identified as an oligodendrocyte-enriched DMG despite being less commonly associated with this lineage. As reference cell type atlases continue to expand, additional unexpected targets may emerge and help refine our understanding of brain cell type-enriched methylation features in TBI.

Our study should be interpreted in the context of several limitations. First, the microglia methylome reference included a limited sample size, which reduces confidence in microglia-enriched interpretations relative to other cell types. In addition, brain injury may alter cellular states, and methylation features may overlap across related glial, immune, and vascular populations. Therefore, the identified features are best interpreted as reference-defined, cell-enriched candidate methylation signatures rather than definitive cell-type-specific markers. Finally, although samples were grouped by post-injury time point, the current dataset is primarily cross-sectional, with only a subset of paired samples. Thus, observations across the 1-day, 1-month, and 6-month groups should be viewed as time point-associated patterns and candidate biomarkers that require confirmation in larger longitudinal cohorts. In sum, these exploratory findings demonstrate the methodology and potential utility of both DMR analysis and deconvolution approaches for interrogating methylomic data. With continued cohort expansion, these complementary tools may help prioritize candidate cfDNA biomarkers and guide future studies of brain cell-associated methylation patterns after TBI.

## Supporting information

S1 File Supplementary materials and methods. (DOCX)

S2 File Supplementary Figure 1. Percentage of sequencing reads overlapping brain cell-enriched DMRs in control and TBI plasma cfDNA samples. (DOCX)

## References

[1] Dhar GA, Saha S, Mitra P, Nag Chaudhuri R. DNA methylation and regulation of gene expression: Guardian of our health. Nucleus (Calcutta). 2021;64(3):259–70. doi: 10.1007/s13237-021-00367-y 34421129 PMC8366481

[2] A DNA methylation atlas of normal human cell types | Nature. [cited 1 Oct 2025]. https://www.nature.com/articles/s41586-022-05580-6 10.1038/s41586-022-05580-6 PMC9811898 36599988

[3] Sahoo K, Sundararajan V. Methods in DNA methylation array dataset analysis: A review. Comput Struct Biotechnol J. 2024;23:2304–25. doi: 10.1016/j.csbj.2024.05.015 38845821 PMC11153885

[4] Akalin A, Kormaksson M, Li S, Garrett-Bakelman FE, Figueroa ME, Melnick A, et al. methylKit: A comprehensive R package for the analysis of genome-wide DNA methylation profiles. Genome Biol. 2012;13(10):R87. doi: 10.1186/gb-2012-13-10-r87 23034086 PMC3491415

[5] Hebestreit K, Dugas M, Klein H-U. Detection of significantly differentially methylated regions in targeted bisulfite sequencing data. Bioinformatics. 2013;29(13):1647–53. doi: 10.1093/bioinformatics/btt263 23658421

[6] Gaspar JM, Hart RP. DMRfinder: Efficiently identifying differentially methylated regions from MethylC-seq data. BMC Bioinformatics. 2017;18(1):528. doi: 10.1186/s12859-017-1909-0 29187143 PMC5817627

[7] Feng H, Wu H. Differential methylation analysis for bisulfite sequencing using DSS. Quant Biol. 2019;7(4):327–34. doi: 10.1007/s40484-019-0183-8 34084562 PMC8171293

[8] Singer BD. A practical guide to the measurement and analysis of DNA methylation. Am J Respir Cell Mol Biol. 2019;61(4):417–28. doi: 10.1165/rcmb.2019-0150TR 31264905 PMC6775954

[9] Lu H, Liu Y, Wang J, Fu S, Wang L, Huang C, et al. Detection of ovarian cancer using plasma cell-free DNA methylomes. Clin Epigenetics. 2022;14(1):74. doi: 10.1186/s13148-022-01285-9 35681212 PMC9185905

[10] Hua X, Zhou H, Wu H-C, Furnari J, Kotidis CP, Rabadan R, et al. Tumor detection by analysis of both symmetric- and hemi-methylation of plasma cell-free DNA. Nat Commun. 2024;15(1):6113. doi: 10.1038/s41467-024-50471-1 39030196 PMC11271492

[11] Houseman EA, Molitor J, Marsit CJ. Reference-free cell mixture adjustments in analysis of DNA methylation data. Bioinformatics. 2014;30(10):1431–9. doi: 10.1093/bioinformatics/btu029 24451622 PMC4016702

[12] Lee D, Lee S, Kim S. PRISM: methylation pattern-based, reference-free inference of subclonal makeup. Bioinformatics. 2019;35(14):i520–9. doi: 10.1093/bioinformatics/btz327 31510697 PMC6612819

[13] Caggiano C, Celona B, Garton F, Mefford J, Black BL, Henderson R, et al. Comprehensive cell type decomposition of circulating cell-free DNA with CelFiE. Nat Commun. 2021;12(1):2717. doi: 10.1038/s41467-021-22901-x 33976150 PMC8113516

[14] Zhang W, Xu H, Qiao R, Zhong B, Zhang X, Gu J, et al. ARIC: Accurate and robust inference of cell type proportions from bulk gene expression or DNA methylation data. Brief Bioinform. 2022;23(1):bbab362. doi: 10.1093/bib/bbab362 34472588

[15] Keukeleire P, Makrodimitris S, Reinders M. Cell type deconvolution of methylated cell-free DNA at the resolution of individual reads. NAR Genom Bioinform. 2023;5(2):lqad048. doi: 10.1093/nargab/lqad048 37274121 PMC10236360

[16] Mukamel EA, Ngai J. Perspectives on defining cell types in the brain. Curr Opin Neurobiol. 2019;56:61–8. doi: 10.1016/j.conb.2018.11.007 30530112 PMC6551297

[17] Ciaunica A, Shmeleva EV, Levin M. The brain is not mental! coupling neuronal and immune cellular processing in human organisms. Front Integr Neurosci. 2023;17:1057622. doi: 10.3389/fnint.2023.1057622 37265513 PMC10230067

[18] Maas AIR, Menon DK, Manley GT, Abrams M, Åkerlund C, Andelic N, et al. Traumatic brain injury: Progress and challenges in prevention, clinical care, and research. Lancet Neurol. 2022;21(11):1004–60. doi: 10.1016/S1474-4422(22)00309-X 36183712 PMC10427240

[19] Kang G, Moo EK, Banton R, Petel OE, Harris AR. Cellular mechanisms of traumatic brain injury. NPJ Biol Phys Mech. 2025;2(1):16. doi: 10.1038/s44341-025-00020-8 40475319 PMC12133593

[20] U.S. Department of Defense. Traumatic Brain Injury: Updated Definition and Reporting. 2015. https://www.health.mil/Reference-Center/Policies/2015/04/06/Traumatic-Brain-Injury-Updated-Definition-and-Reporting

[21] Krueger F, Andrews SR. Bismark: A flexible aligner and methylation caller for Bisulfite-Seq applications. Bioinformatics. 2011;27(11):1571–2. doi: 10.1093/bioinformatics/btr167 21493656 PMC3102221

[22] Heller G, Topakian T, Altenberger C, Cerny-Reiterer S, Herndlhofer S, Ziegler B, et al. Next-generation sequencing identifies major DNA methylation changes during progression of Ph+ chronic myeloid leukemia. Leukemia. 2016;30(9):1861–8. doi: 10.1038/leu.2016.143 27211271 PMC5240019

[23] Xu X, Elkenani M, Tan X, Hain J, Cui B, Schnelle M. DNA methylation analysis identifies novel epigenetic loci in dilated murine heart upon exposure to volume overload. Int J Mol Sci. 2023;24:5885. doi: 10.3390/ijms24065885 PMC10059258 36982963

[24] Ge SX, Jung D, Yao R. ShinyGO: A graphical gene-set enrichment tool for animals and plants. Bioinformatics. 2020;36(8):2628–9. doi: 10.1093/bioinformatics/btz931 31882993 PMC7178415

[25] Snyder MW, Kircher M, Hill AJ, Daza RM, Shendure J. Cell-free DNA comprises an in vivo nucleosome footprint that informs its tissues-of-origin. Cell. 2016;164(1–2):57–68. doi: 10.1016/j.cell.2015.11.050 26771485 PMC4715266

[26] Moss J, Magenheim J, Neiman D, Zemmour H, Loyfer N, Korach A, et al. Comprehensive human cell-type methylation atlas reveals origins of circulating cell-free DNA in health and disease. Nat Commun. 2018;9(1):5068. doi: 10.1038/s41467-018-07466-6 30498206 PMC6265251

[27] Lui YYN, Chik K-W, Chiu RWK, Ho C-Y, Lam CWK, Lo YMD. Predominant hematopoietic origin of cell-free DNA in plasma and serum after sex-mismatched bone marrow transplantation. Clin Chem. 2002;48(3):421–7. doi: 10.1093/clinchem/48.3.421 11861434

[28] Peferoen L, Kipp M, van der Valk P, van Noort JM, Amor S. Oligodendrocyte-microglia cross-talk in the central nervous system. Immunology. 2014;141(3):302–13. doi: 10.1111/imm.12163 23981039 PMC3930369

[29] Kalafatakis I, Karagogeos D. Oligodendrocytes and microglia: Key players in myelin development, damage and repair. Biomolecules. 2021;11:1058. doi: 10.3390/biom11071058 PMC8301746 34356682

[30] Watkins S, Robel S, Kimbrough IF, Robert SM, Ellis-Davies G, Sontheimer H. Disruption of astrocyte-vascular coupling and the blood-brain barrier by invading glioma cells. Nat Commun. 2014;5:4196. doi: 10.1038/ncomms5196 24943270 PMC4127490

[31] Cabezas R, Avila M, Gonzalez J, El-Bachá RS, Báez E, García-Segura LM, et al. Astrocytic modulation of blood brain barrier: Perspectives on Parkinson’s disease. Front Cell Neurosci. 2014;8:211. doi: 10.3389/fncel.2014.00211 25136294 PMC4120694

[32] Gong Y, Wu M, Huang Y, He X, Yuan J, Dang B. Research developments in the neurovascular unit and the blood‑brain barrier (Review). Biomed Rep. 2025;22(5):88. doi: 10.3892/br.2025.1966 40166412 PMC11956146

[33] Chung CY, Koprich JB, Hallett PJ, Isacson O. Functional enhancement and protection of dopaminergic terminals by RAB3B overexpression. Proc Natl Acad Sci U S A. 2009;106(52):22474–9. doi: 10.1073/pnas.0912193106 20007772 PMC2791667

[34] Pietrobon D, Conti F. Astrocytic Na+, K+ ATPases in physiology and pathophysiology. Cell Calcium. 2024;118:102851. doi: 10.1016/j.ceca.2024.102851 38308916

[35] Joseph UG, Oyovwi MO, Jeroh E, Esuku DT, Ben-Azu B. Dysfunctional astrocyte metabolism: A driver of imbalanced excitatory/inhibitory tone and support for therapeutic intervention targets. J Mol Pathol. 2025;6:12. doi: 10.3390/jmp6020012

[36] Mariani MM, Kielian T. Microglia in infectious diseases of the central nervous system. J Neuroimmune Pharmacol. 2009;4(4):448–61. doi: 10.1007/s11481-009-9170-6 19728102 PMC2847353

[37] Simons M, Nave KA. Oligodendrocytes: Myelination and Axonal Support. Cold Spring Harb Perspect Biol. 2016;8:a020479. doi: 10.1101/cshperspect.a020479 PMC4691794 26101081

[38] Philips T, Rothstein JD. Oligodendroglia: Metabolic supporters of neurons. J Clin Invest. 2017;127(9):3271–80. doi: 10.1172/JCI90610 28862639 PMC5669561

[39] Stamatovic SM, Johnson AM, Keep RF, Andjelkovic AV. Junctional proteins of the blood-brain barrier: New insights into function and dysfunction. Tissue Barriers. 2016;4(1):e1154641. doi: 10.1080/21688370.2016.1154641 27141427 PMC4836471

[40] Krolak T, Kaplan L, Navas K, Chen L, Birmingham A, Ryvkin D, et al. Brain endothelial gap junction coupling enables rapid vasodilation propagation during neurovascular coupling. Cell. 2025;188(18):5003-5019.e22. doi: 10.1016/j.cell.2025.06.030 40675149 PMC12337775

[41] Stuart T, Butler A, Hoffman P, Hafemeister C, Papalexi E, Mauck WM, et al. Comprehensive integration of single-cell data. Cell. 2019;177(7):1888-1902.e21. doi: 10.1016/j.cell.2019.05.031 31178118 PMC6687398

[42] Guo JU, Ma DK, Mo H, Ball MP, Jang M-H, Bonaguidi MA, et al. Neuronal activity modifies the DNA methylation landscape in the adult brain. Nat Neurosci. 2011;14(10):1345–51. doi: 10.1038/nn.2900 21874013 PMC3183401

[43] Moore LD, Le T, Fan G. DNA methylation and its basic function. Neuropsychopharmacology. 2013;38:23–38. doi: 10.1038/npp.2012.112 PMC3521964 22781841

[44] Spainhour JC, Lim HS, Yi SV, Qiu P. Correlation patterns between DNA methylation and gene expression in the cancer genome atlas. Cancer Informatics. 2019;18:1176935119828776. doi: 10.1177/1176935119828776 PMC6376553 30792573

[45] Zhang Z-Y, Zhang Z, Fauser U, Schluesener HJ. Global hypomethylation defines a sub-population of reactive microglia/macrophages in experimental traumatic brain injury. Neurosci Lett. 2007;429(1):1–6. doi: 10.1016/j.neulet.2007.09.061 17996371

[46] Lundberg J, Karimi M, von Gertten C, Holmin S, Ekström TJ, Sandberg-Nordqvist A-C. Traumatic brain injury induces relocalization of DNA-methyltransferase 1. Neurosci Lett. 2009;457(1):8–11. doi: 10.1016/j.neulet.2009.03.105 19429151

[47] Bailey ZS, Grinter MB, De La Torre Campos D, VandeVord PJ. Blast induced neurotrauma causes overpressure dependent changes to the DNA methylation equilibrium. Neurosci Lett. 2015;604:119–23. doi: 10.1016/j.neulet.2015.07.035 26232681

[48] Sagarkar S, Bhamburkar T, Shelkar G, Choudhary A, Kokare DM, Sakharkar AJ. Minimal traumatic brain injury causes persistent changes in DNA methylation at BDNF gene promoters in rat amygdala: A possible role in anxiety-like behaviors. Neurobiol Dis. 2017;106:101–9. doi: 10.1016/j.nbd.2017.06.016 28663119

[49] Adel SS, Pranske ZJ, Kowalski TF, Kanzler N, Ray R, Carmona C, et al. Plexin-B1 and Plexin-B2 play non-redundant roles in GABAergic synapse formation. Mol Cell Neurosci. 2024;128:103920. doi: 10.1016/j.mcn.2024.103920 38331011 PMC11046529

