# Supplementary material for: CfDNA methylation profiling in blood following moderate-to-severe traumatic brain injury using brain cell type–enriched signatures: A pilot study
Source: PLoS One. 2026 Oct 6;21(10):e0358763. doi: 10.1371/journal.pone.0358763 (PMC13641372; doi:10.1371/journal.pone.0358763)
Supplement: Supplementary file 1 [file pone.0358763.s001.docx]

**Supplementary Materials and Methods**

**Patients.** Patients included this study were enrolled in the DOD program "Gap-Based Milieu Biomarkers for Traumatic Brain Injury (GAMBIT-TBI)" (Award # W81XWH2211089). Standardized case report forms were developed and collected from each individual including demographics, injury characteristics, and outcomes.

Samples collected at 1-day post-injury (N = 4) were obtained exclusively from Baylor College of Medicine (BCM), with time after injury between 9-22 hours.

1-month post-injury samples (N = 12) were obtained exclusively from University of Pittsburgh (UPitt), with time after injury in a range between 13 days to 107 days post-injury, with most samples collected between 21-60 days post-injury (median = 43 days).

6-month post-injury samples (N = 4) were also exclusively obtained from UPitt, with time after injuries of 176-189 days post-injury (median = 184 days).

Uninjured healthy control samples (N = 4) were collected from UPitt. One additional healthy control cfDNA was obtained from Biochain (Newark, CA), for a total of N = 5 controls.

Each cohort description is as follows:

UPitt Cohort Description

*Inclusion & exclusion criteria:* Patients were included if they met the following criteria: non-penetrating closed head injury verified with TBI related ICD-9 diagnosis code and/or sufficient medical documentation of medical or functional complications, such as positive anatomic neuroimaging findings or focal neurological signs on day of injury. Individuals must be at least 18 years of age or older, and use English as primary language to be eligible for inclusion. Penetrating head injuries, participants under 18 years of age, participants over 79 years of age, untreated endocrine disorders, autoimmune disorders, history of significant neurological or neurodegenerative disease, documented history of previous TBI or stroke, and incarceration were the exclusion criteria. Participants enrolled as control subjects were included if: ages 18-79, had no prior history of TBI or stroke, no history of endocrine dysfunction, no history of autoimmune disorders, no history of significant neurological or neurodegenerative disease, and use English as primary language. All participants or participant's next of kin provided written informed consent prior to enrollment in study activities.

*Clinical variable collection:* Demographics including age, sex, race, education level, sample collection time points, and injury severity variables, such as best GCS in first 24 hours, mechanism of injury and acute CT findings, were included (Supplementary Table 1).

BCM Cohort Description

*Inclusion & exclusion criteria*: Patients were included if they met the following criteria: (1) Closed TBI, (2) Age greater than 18 years old, (3) GCS 3-12 or GCS 13-15 with positive or negative CT head scan, (4) Primary language English or Spanish, (5) Enrolled within 24hrs of injury. Penetrating injuries, life threatening polytrauma, associated spinal cord injury and severe pre-existing diseases were the exclusion criteria. Samples were obtained after signed consent form either by the patient or the legally authorized representative (LAR) according to local protocol. A 3-day waiver of consent was granted for the first blood samples pending LAR identification according to local protocol.

*Clinical variable collection:* Demographics including age, sex, race, education level, sample collection time points, and injury severity variables, such as best GCS in first 24 hours, mechanism of injury and acute CT findings, were included (Supplementary Table 1).
