# Supplementary material for: CfDNA methylation profiling in blood following moderate-to-severe traumatic brain injury using brain cell type–enriched signatures: A pilot study
Source: PLoS One. 2026 Oct 6;21(10):e0358763. doi: 10.1371/journal.pone.0358763 (PMC13641372; doi:10.1371/journal.pone.0358763)
Supplement: Supplementary file 2 [file pone.0358763.s002.docx]

**Supplementary Figure 1. Percentage of sequencing reads overlapping brain cell-enriched DMRs in control and TBI plasma cfDNA samples.** The number of reads overlapping brain cell-enriched DMRs was normalized to the total number of uniquely mapped reads after sequencing data processing.
