# Supplementary material for: CfDNA methylation profiling in blood following moderate-to-severe traumatic brain injury using brain cell type–enriched signatures: A pilot study
Source: PLoS One. 2026 Oct 6;21(10):e0358763. doi: 10.1371/journal.pone.0358763 (PMC13641372; doi:10.1371/journal.pone.0358763)
Supplement: Supplementary file 3 [file pone.0358763.s004.docx]

We thank the editor and reviewers for their thoughtful and constructive comments. We have carefully revised the manuscript and addressed all points raised. Below please find our point-by-point responses:

Journal Requirements:

When submitting your revision, we need you to address these additional requirements.

***1.Please ensure that your manuscript meets PLOS ONE's style requirements, including those for file naming. The PLOS ONE style templates can be found at***

***https://journals.plos.org/plosone/s/file?id=wjVg/PLOSOne_formatting_sample_main_body.pdf and***

[***https://journals.plos.org/plosone/s/file?id=ba62/PLOSOne_formatting_sample_title_authors_affiliations.pdf***](https://journals.plos.org/plosone/s/file?id=ba62/PLOSOne_formatting_sample_title_authors_affiliations.pdf)

**Response:** We checked the hyperlink and revised the manuscript to comply with the journal’s formatting and style requirements.

***2. Please note that PLOS One has specific guidelines on code sharing for submissions in which author-generated code underpins the findings in the manuscript. In these cases, we expect all author-generated code to be made available without restrictions upon publication of the work. Please review our guidelines at https://journals.plos.org/plosone/s/materials-and-software-sharing#loc-sharing-code and ensure that your code is shared in a way that follows best practice and facilitates reproducibility and reuse.***

**Response:** We thank the Editor for highlighting the PLOS ONE code-sharing requirements. The analyses in this study were performed using established, publicly available software packages and standard analysis pipelines. No custom or author-generated code was developed that underpins the findings of the manuscript; therefore, there is no author-generated code to share.

***3. We note that the grant information you provided in the ‘Funding Information’ and ‘Financial Disclosure’ sections do not match.***

***When you resubmit, please ensure that you provide the correct grant numbers for the awards you received for your study in the ‘Funding Information’ section.***

**Response:** We thank the Editor for noting this discrepancy. We removed the funding statement from the manuscript and updated the “Financial Disclosure” section in the submission system to ensure that the funding information is accurate and consistent.

***4. Thank you for stating the following financial disclosure:***

***“This study was supported by the CHOP Resuscitation Science Center and the Department of Defense (DoD). Effort sponsored by the Government under Focused Program Award Number W81XWH-22-1-1089 (RR).”***

***Please state what role the funders took in the study.  If the funders had no role, please state: "The funders had no role in study design, data collection and analysis, decision to publish, or preparation of the manuscript."***

***If this statement is not correct you must amend it as needed.***

***Please include this amended Role of Funder statement in your cover letter; we will change the online submission form on your behalf.***

**Response:** We thank the Editor for this clarification. We updated the Financial Disclosure section in the submission system to include the required Department of Defense disclaimer. We also added the following Role of Funder statement to the cover letter: “The funders had no role in study design, data collection and analysis, decision to publish, or preparation of the manuscript.”

***5. Please note that funding information should not appear in any section or other areas of your manuscript. We will only publish funding information present in the Funding Statement section of the online submission form. Please remove any funding-related text from the manuscript.***

**Response:** We have removed all funding-related text from the manuscript, as requested.

***6. Please note that your Data Availability Statement is currently missing the repository name and/or the DOI/accession number of each dataset OR a direct link to access each database. If your manuscript is accepted for publication, you will be asked to provide these details on a very short timeline. We therefore suggest that you provide this information now, though we will not hold up the peer review process if you are unable.***

**Response:** We thank the Editor for this clarification. The sequencing data are part of an ongoing study and have not yet been deposited in a public repository. As originally outlined in our grant, we planned to deposit the data in the Federal Interagency Traumatic Brain Injury Research (FITBIR) Informatics System. However, we were recently informed that FITBIR may no longer be available as a repository for data submission. We are seeking further clarification regarding its status and simultaneously evaluating appropriate alternative repositories for data deposition and sharing. We remain committed to depositing and sharing the sequencing data through a suitable public repository.

***7. Please remove your figures from within your manuscript file, leaving only the individual TIFF/EPS image files, uploaded separately. These will be automatically included in the reviewers’ PDF.***

**Response:** We have removed all figures from the manuscript file and uploaded each figure separately in TIFF format, as requested.

***8. We note that there is identifying data in the Supporting Information file “Supplementary Table 1.xlsx” Due to the inclusion of these potentially identifying data, we have removed this file from your file inventory. Prior to sharing human research participant data, authors should consult with an ethics committee to ensure data are shared in accordance with participant consent and all applicable local laws.***

***Data sharing should never compromise participant privacy. It is therefore not appropriate to publicly share personally identifiable data on human research participants. The following are examples of data that should not be shared:***

***-Name, initials, physical address***

***-Ages more specific than whole numbers***

***-Internet protocol (IP) address***

***-Specific dates (birth dates, death dates, examination dates, etc.)***

***-Contact information such as phone number or email address***

***-Location data***

***-ID numbers that seem specific (long numbers, include initials, titled “Hospital ID”) rather than random (small numbers in numerical order)***

***Data that are not directly identifying may also be inappropriate to share, as in combination they can become identifying. For example, data collected from a small group of participants, vulnerable populations, or private groups should not be shared if they involve indirect identifiers (such as sex, ethnicity, location, etc.) that may risk the identification of study participants.***

***Additional guidance on preparing raw data for publication can be found in our Data Policy (https://journals.plos.org/plosone/s/data-availability#loc-human-research-participant-data-and-other-sensitive-data) and in the following article: http://www.bmj.com/content/340/bmj.c181.long.***

***Please remove or anonymize all personal information (<specific identifying information in file to be removed>), ensure that the data shared are in accordance with participant consent, and re-upload a fully anonymized data set. Please note that spreadsheet columns with personal information must be removed and not hidden as all hidden columns will appear in the published file.***

***Response:*** We thank the Editor for bringing this concern to our attention. To fully address the issue and ensure compliance with PLOS ONE’s guidelines regarding identifiable data, we have removed S1 Table from the revised manuscript.

***9. Please include captions for your Supporting Information files at the end of your manuscript, and update any in-text citations to match accordingly. Please see our Supporting Information guidelines for more information:*** [***http://journals.plos.org/plosone/s/supporting-information***](http://journals.plos.org/plosone/s/supporting-information)***.***

**Response:** We have added a Supporting Information section containing captions for all Supporting Information files at the end of the manuscript. We have also revised the corresponding in-text citations to ensure that they match the file labels.

***10. If the reviewer comments include a recommendation to cite specific previously published works, please review and evaluate these publications to determine whether they are relevant and should be cited. There is no requirement to cite these works unless the editor has indicated otherwise.***

**Response:** Reviewers did not recommend citing any specific previously published works.

***11. Please review your reference list to ensure that it is complete and correct. If you have cited papers that have been retracted, please include the rationale for doing so in the manuscript text, or remove these references and replace them with relevant current references. Any changes to the reference list should be mentioned in the rebuttal letter that accompanies your revised manuscript. If you need to cite a retracted article, indicate the article’s retracted status in the References list and also include a citation and full reference for the retraction notice.***

**Response:** We have carefully reviewed the reference list and determined that no changes are necessary.

Additional Editor Comments:

Dear Authors, 
The reviewers are enthusiastic about your submission. We hope you are able to respond to the reviewers.
Sincerely,
Nafisa

**Comments to the Author**

***1. Is the manuscript technically sound, and do the data support the conclusions?

The manuscript must describe a technically sound piece of scientific research with data that supports the conclusions. Experiments must have been conducted rigorously, with appropriate controls, replication, and sample sizes. The conclusions must be drawn appropriately based on the data presented.***

***Reviewer #1: Yes***

***Reviewer #2: Yes***

**Response:** We thank the reviewer for the positive feedback and for recognizing the strengths of this study.

***2. Has the statistical analysis been performed appropriately and rigorously?***

***Reviewer #1: Yes***

***Reviewer #2: Yes***

**Response:** We thank the reviewer for the positive feedback and for recognizing the strengths of this study.

***3. Have the authors made all data underlying the findings in their manuscript fully available?

The*** [***PLOS Data policy***](http://www.plosone.org/static/policies.action#sharing) ***requires authors to make all data underlying the findings described in their manuscript fully available without restriction, with rare exception (please refer to the Data Availability Statement in the manuscript PDF file). The data should be provided as part of the manuscript or its supporting information, or deposited to a public repository. For example, in addition to summary statistics, the data points behind means, medians and variance measures should be available. If there are restrictions on publicly sharing data—e.g. participant privacy or use of data from a third party—those must be specified.***

***Reviewer #1: No***

***Reviewer #2: Yes***

**Response:** We thank the Editor for this clarification. The sequencing data are part of an ongoing study and have not yet been deposited in a public repository. As originally outlined in our grant, we planned to deposit the data in the Federal Interagency Traumatic Brain Injury Research (FITBIR) Informatics System. However, we were recently informed that FITBIR may no longer be available as a repository for data submission. We are seeking further clarification regarding its status and simultaneously evaluating appropriate alternative repositories for data deposition and sharing. We remain committed to depositing and sharing the sequencing data through a suitable public repository.

***4. Is the manuscript presented in an intelligible fashion and written in standard English?

PLOS ONE does not copyedit accepted manuscripts, so the language in submitted articles must be clear, correct, and unambiguous. Any typographical or grammatical errors should be corrected at revision, so please note any specific errors here.***

***Reviewer #1: Yes***

***Reviewer #2: Yes***

**Response:** We thank the reviewer for the positive feedback and for recognizing the strengths of this study.

***5. Review Comments to the Author

Please use the space provided to explain your answers to the questions above. You may also include additional comments for the author, including concerns about dual publication, research ethics, or publication ethics. (Please upload your review as an attachment if it exceeds 20,000 characters)***

***Reviewer #1: In the manuscript, the researchers are utilizing broad and contemporary methylomic methodologies to explore cfDNA cell-type deconvolution in TBI patients. The bioinformatic approach and framework have a lot of utility and are biologically informative. The authors were able to identify a few notable methylation signatures using the differential methylation region analysis to identify major cell classes and detect differences between TBI patients and control patients. The authors are cautious with their interpretations and suggest that the current study can help to justify further work in the area. There are some notable limitations that don't necessarily cause issue but are worth having further clarified to avoid any overgeneralization. There is a limited sample (n of 1) for the microglia cell datasets, which limits the statistical robustness of the interpretations being made. Furthermore, brain injury likely changes cell states, and methylation signatures can overlap biologically, suggesting that while the data do support cell-enriched signatures, it may be an overstatement to say that they support truly cell-type-specific signatures. The authors also state different temporal interpretations with different types of trajectories, but care is needed here as the dataset is largely cross-sectional rather than a true longitudinal dataset, with a progression that would be if the data were repeatedly collected from the same individuals. This is not to say that the use of cross-sectional data is inappropriate, just that caution needs to be taken when discussing the data longitudinally. The authors should reference the data more as potential candidate biomarkers to avoid readers concluding that these are validated biomarkers, as the data is early in discovery. Caution should also be taken with any mechanistic or pathophysiological interpretations, as they may exceed the evidence being presented. Especially considering that the study is fundamentally descriptive in nature. Overall, this study is rather novel in its examination of cfDNA following TBI. The authors have a promising manuscript that should tone down the mechanistic explanations and focus on the potential directions that the research can help to direct future studies.***

**Response:** We thank the reviewer for their thoughtful and constructive assessment of the manuscript and for recognizing the utility and novelty of our methylomic framework for studying cfDNA in TBI. We agree that the current study is exploratory and descriptive in nature and that the findings should be interpreted as hypothesis-generating rather than as validated biomarkers or definitive mechanistic evidence.

In response to this comment, we revised the manuscript throughout to avoid overgeneralization. Specifically, we replaced language referring to “cell-type-specific” signatures with more cautious wording such as “cell type-enriched,” “cell-associated,” and “reference-defined cell type–enriched” methylation features. We also revised the **Abstract**, **Results**, and **Discussion** to describe the identified DMGs as candidate methylation markers or candidate biomarkers that require future validation.

We also agree with the reviewer that the limited microglia reference dataset reduces the statistical robustness of microglia-related interpretations. To address this, we added a dedicated limitations paragraph noting that the microglia methylome reference included a limited sample size and that methylation features may overlap across related glial, immune, and vascular populations, especially in the setting of injury-associated changes in cell state.

In addition, we revised the temporal language throughout the manuscript. Because the human cfDNA dataset is primarily cross-sectional, with only a subset of paired samples, we no longer describe the findings as definitive longitudinal trajectories or progression patterns. Instead, we now refer to these findings as post-injury timepoint-associated methylation patterns observed across 1-day, 1-month, and 6-month sampling groups.

Finally, we toned down mechanistic and pathophysiological interpretations in the **Abstract** and **Discussion**. We now frame the gene-level and pathway findings as biologically plausible, rather than as evidence of causal mechanisms. Together, these revisions clarify that this pilot study supports the feasibility of using cfDNA methylation profiling to nominate candidate brain cell–associated methylation signatures in TBI, while emphasizing the need for larger longitudinal cohorts and independent validation.

***Reviewer #2: Thank you for the editor's invitation. This is an innovative and exploratory preliminary study, representing the first attempt to use brain cell type specific DNA methylation features to determine the cellular origin of plasma cfDNA from patients with moderate to severe TBI at multiple time points. The authors established a methylation reference atlas for five major brain cell types and demonstrated the potential of this approach for tracking dynamic changes in brain cell injury after TBI in a small scale clinical cohort. However, the current version has several critical limitations, particularly regarding sample size, statistical power, methodological validation, rigor of data interpretation, and the alignment between results and conclusions. The following issues need to be carefully addressed by the authors in a major revision.
1. The methylation changes shown as "increases" or "decreases" in Figures 2F, 3F, etc., are presented without any statistical tests (e.g., p values or confidence intervals). The authors should clearly state that these are only descriptive observations and cannot be interpreted as statistically significant differences. Throughout the manuscript, phrases such as "showed larger increases" should be replaced with more cautious wording, e.g., "suggested a trend of increase."***

**Response:** We thank the reviewer for this important comment. We agree that, in the absence of formal statistical testing, the observed methylation differences should not be interpreted as statistically significant. We have therefore revised the manuscript throughout to tone down the language and clearly present these findings as descriptive observations. Statements such as “showed larger increases” or “showed the greatest reduction” have been replaced with more cautious wording, including “suggested a trend toward reduced methylation” and “appeared to exhibit higher methylation levels.” We have also clarified that these patterns are exploratory and require confirmation in larger cohorts with appropriate statistical testing.

***2. The proportion of brain derived cfDNA in plasma cfDNA is typically very low (<1%). The authors did not provide any data on the percentage of reads mapped to brain cell specific DMRs relative to total reads in control and TBI samples. This information should be added.***

**Response:** We thank the reviewer for this important comment. We calculated the percentage of reads overlapping brain cell-enriched DMRs relative to the total number of uniquely mapped reads after sequencing processing. The proportion was comparable between TBI samples (median, 0.248%; IQR, 0.233%–0.255%) and control samples (median, 0.250%; IQR, 0.238%–0.254%). These data have now been added as Supplementary Figure 1 and described in the revised manuscript.

We note that the proportion of reads overlapping brain cell-enriched DMRs reflects sequencing coverage of these genomic regions and should not, by itself, be interpreted as the fraction of brain-derived cfDNA. Because the same genomic loci are present in cfDNA originating from multiple tissues, identification of a brain-enriched cfDNA signal additionally depends on whether the fragments exhibit the expected brain-associated methylation pattern. We have clarified this distinction in the revised manuscript.

***3. In the ethics statement, the recruitment end date for UPitt is given as September 3, 2025 (a future date?), while the manuscript submission date is 2026? Please verify. This may be a typographical error.***

**Response:** We appreciate the reviewer’s comment. The recruitment end date of September 3, 2025 stated in the ethics statement is accurate and not an error. This date reflects the point at which active enrollment of new subjects at the University of Pittsburgh site was closed. Recruitment of subjects has concluded as of that date; the study has since transitioned to its follow-up phase, during which only scheduled follow-up visits with already-enrolled participants are being conducted. No new subjects are being recruited. This explains why the manuscript submission date in 2026 postdates the recruitment end date—the intervening period reflects ongoing follow-up data collection and manuscript preparation.

***6. PLOS authors have the option to publish the peer review history of their article (***[***what does this mean?***](https://journals.plos.org/plosone/s/editorial-and-peer-review-process#loc-peer-review-history)***). If published, this will include your full peer review and any attached files.***
 ***If you choose “no”, your identity will remain anonymous but your review may still be made public.

Do you want your identity to be public for this peer review? For information about this choice, including consent withdrawal, please see our*** [***Privacy Policy***](https://www.plos.org/privacy-policy)***.***

***Reviewer #1: Yes: Michael J Hylin***

***Reviewer #2: Yes: Xuanjie Chen***

**Response:** No. We do not consent to having our identity made public in connection with this peer review.
